# Treatment satisfaction and quality-of-life between type 2 diabetes patients initiating long- vs. intermediate-acting basal insulin therapy in combination with oral hypoglycemic agents – a randomized, prospective, crossover, open clinical trial

**DOI:** 10.1186/s12955-015-0279-4

**Published:** 2015-06-09

**Authors:** Norbert Hermanns, Bernd Kulzer, Thomas Kohlmann, Stephan Jacob, Wolfgang Landgraf, Karlheinz Theobald, Thomas Haak

**Affiliations:** Forschungsinstitut der Diabetes Akademie Bad Mergentheim (FIDAM), Theodor-Klotzbücher-Str. 12, 97980 Bad Mergentheim, Germany; Institute for Community Medicine, Greifswald, Germany; Private Practice, Villingen-Schwenningen, Germany; Sanofi, Frankfurt, Germany

**Keywords:** Diabetes, Insulin glargine, NPH insulin, Health assessment, Treatment satisfaction, Quality of life

## Abstract

**Background:**

Pharmacological and clinical differences between insulin glargine and NPH insulin may translate into differences in patient reported outcomes, but existing data are equivocal.

**Methods:**

In this 48-week, open-label, randomized, multi-center, crossover phase IV trial, insulin naïve type 2 diabetes patients with blood glucose not at target on oral hypoglycemic agents had basal insulin added to their treatment regimen. A total of 343 patients were randomized to either receive insulin glargine (n = 176; sequence A) or neutral protamine Hagedorn (NPH) insulin (n = 167; sequence B) in period 1 (weeks 1–24) and vice versa in period 2 (weeks 25–48). The primary objective was to assess patient reported outcomes using a composite Diabetes Related Quality of Life (DRQoL) score based on an unweighted Insulin Treatment Experience Questionnaire (ITEQ) score, a Problem Areas in Diabetes (PAID) questionnaire score, and the mental health score in the Short Form (SF)-12® Health Survey, analyzed by analysis of covariance (ANCOVA).

**Results:**

Patients (mean age 62.3 ± 9.0; 39.5 % female) had a mean diabetes duration of 9.6 ± 5.9 years, a mean baseline HbA1c of 8.15 ± 0.72 %, and a mean fasting blood glucose (FBG) level of 9.37 ± 2.19 mmol/L. A total of 229 patients were available for primary endpoint evaluation (modified intention to treat population). Combining all data from both periods for each insulin treatment, on a 0–100 scale, the mean DRQoL score was 69.6 (±9.04) with insulin glargine and 70.0 (±9.40) with NPH insulin. Neither an effect of treatment with insulin glargine vs NPH insulin (p = 0.31) nor a period effect (p = 0.96), nor a sequence effect (p = 0.76) was observed using ANCOVA.

**Conclusions:**

The results show that in a patient population with sub-optimal glycemic control at baseline, and a low target achievement rate together with a low rate of hypoglycemia, differences in the patient reported outcomes evaluated in this study were negligible between insulin glargine and NPH insulin.

**Trial registration:**

Clinicaltrials.gov identifier: NCT00941369

## Background

It is well-established that achieving adequate glycemic control reduces the risk of cardiovascular complications in patients with type 2 diabetes [1, 2]. While oral antihyperglycemic treatments are often sufficient for reducing blood glucose levels in newly diagnosed patients, the progressive nature of the disease eventually requires insulin to be added to maintain glycemic control [3]. Initiation of such treatment can either proceed by administration of insulin as a monotherapy, or by the addition of long-acting insulins such as neutral protamine Hagedorn (NPH) insulin or insulin glargine to the oral regimen (basal insulin supported oral therapy; BOT) [4, 5].

While NPH insulin has been shown to effectively reduce blood glucose levels, its peak in activity at around 4–6 h after administration can result in hypoglycemia [6–8]. In comparison, insulin glargine has a much smoother activity profile, resulting in a lower incidence of hypoglycemic events [6, 7, 9–11]. Another important factor to be taken into account when initiating BOT is the duration of NPH activity of only 12–16 h, often resulting in the necessity of twice daily injections. In contrast, the prolonged activity of insulin glargine means that a single daily injection is sufficient for maintaining adequate glucose control. Since risk of hypoglycemia, reduction in lifestyle flexibility, weight gain, and management of injections all have been reported to contribute to the reluctance of patients to take insulin (psychological insulin resistance), the named differences between these two insulin regimens could be significant [12–14].

There are only two studies to date that included an investigation of treatment satisfaction when adding insulin glargine versus NPH insulin to oral therapy in patients with type 2 diabetes. Both, Eliaschewitz *et al.* and Witthaus *et al.* demonstrated slightly higher treatment satisfaction with insulin glargine (p < 0.02 and p = 0.0634, respectively) [8, 15]. Observational data (LIVE-DE) retrospectively taken from questionnaires regarding insulin treatment satisfaction and psychological impact has also indicated a trend towards improved quality of life for patients receiving insulin glargine in comparison to NPH insulin [16]. However, it is clear that further study is required in order to clarify the differences between these two treatments.

Therefore, the primary objective of this study was to investigate the impact of insulin glargine versus NPH insulin on a composite Diabetes Related Quality of Life score (DRQoL), consisting of a standardized and unweighted Insulin Treatment Experience Questionnaire Score (ITEQ), a Problem Areas in Diabetes (PAID) questionnaire score, and the mental health score in the Short Form (SF)-12® Health Survey, in a randomized controlled study. The specific combination of these scores allows for evaluation of a wide variety of PROs, including general quality of life, diabetes-related emotional distress, and overall mental health.

## Patients and methods

The investigation reported here was a 51-week (2 weeks of screening, 2 × 24 weeks treatment, 1 week follow-up), open-label, randomized, multi-center, crossover phase IV trial in insulin naïve type 2 diabetes patients with insufficient metabolic control and HbA1c values ≥7.0 % and ≤10.0 % (to decrease the likely need for prandial insulin supplementation) despite treatment with oral hypoglycemic agents (OHAs). The study protocol was conducted in accordance with good clinical practice and the Declaration of Helsinki, was approved by the Ethics Committee of the Landesärztekammer Baden-Württemberg (Stuttgart, Germany) on May 19th 2009, and registered with clinicaltrials.gov (NCT00941369). All patients provided written informed consent prior to inclusion.

### Patient selection

Patients of either gender (aged 18–80 years) with type 2 diabetes mellitus according to the American Diabetes Association criteria [17], were considered eligible for the study. Further inclusion criteria were a body mass index (BMI) of >22 to <40 kg/m^2^, HbA1c of ≥7.0 to ≤10.0 %, and fasting blood glucose (FBG) of ≥120 mg/dL (6.7 mmol/L).

In order to allow for an accurate comparison of the two types of insulin, patients were excluded from the study if they had received treatment with any insulin within the 3 months prior to inclusion, treatment with more than two OHAs within the 4 weeks prior to inclusion, or continuous treatment with thiazolidinediones or glucagon-like peptide (GLP)-1 receptor agonists. Other factors that may significantly affect quality of life or emotional well-being were also indications for exclusion from the study. These included a history of ketoacidosis, a history of drug or alcohol abuse, diabetic retinopathy with surgical treatment (laser photocoagulation or vitrectomy) in the 3 months prior to study entry or which may require surgical treatment within 3 months, prior pancreatectomy, impaired hepatic function, impaired renal function, current treatment for a psychiatric illness (not further specified), systemic corticoid treatment for more than 2 months, prior bariatric surgery, or major dietary changes for weight management during the last 3 months resulting in weight reduction of >5 kg.

### Study design and treatments

**Fig. 1:**
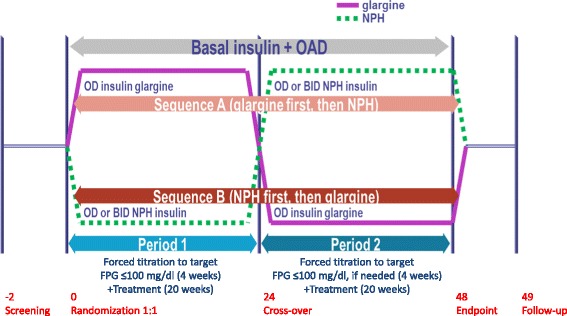
Study design. OAD, oral antidiabetic drugs / oral hypoglycemic agents corresponding to a maximum of 2 drugs out of metformin, sulfonylurea or a DPP-IV inhibitor; NPH, neutral protamine Hagedorn

The study consisted of a two-week screening phase, followed by two 24-week treatment periods, without a washout period in between. After the second treatment period, patients were followed for an additional week (Fig. 1). In each study center, patients were block randomized on a 1:1 basis to either sequence A, starting with insulin glargine (period 1; weeks 1–24) and then switching to NPH insulin (period 2; weeks 25–48); or to sequence B, starting with NPH insulin (period 1) and then switching to insulin glargine (period 2). A crossover design was chosen to allow for patients to serve as their own controls.

Insulin glargine (Sanofi, Berlin, Germany) and NPH insulin (Sanofi, Berlin, Germany) were injected with the TactiPen® injector pen (Sanofi, Berlin, Germany), which is a re-usable insulin delivery device. Insulin glargine was administered by subcutaneous injection once daily, at any time, but each day at the same time. NPH basal insulin was administered at bedtime (21:00–23:00). If the NPH dose was exceeding 30 IE and/or nocturnal hypoglycaemia occured, the NPH dose was split into two doses. One dose was injected at bedtime as described and the second dose was given in the morning (07:00 – 09:00).

During the first week of the treatment phase (forced titration phase), insulin titration was carried out daily. The titration target value was FBG ≤ 5.6 mmol/L. Patients increased their insulin dose following a predefined titration algorithm until the target FBG value was reached. The starting doses were 10 units (U) of insulin glargine and 10 insulin units (I.U.) of NPH insulin per day. At 24 weeks, patients were switched to their second insulin treatment regimen, again following the predefined titration schedule. Four-week titration schemes were used to obtain the same glycemic treatment targets in both insulin therapy regimens during each treatment period, a procedure which was only limited by general limitations of diabetes treatment, such as increasing hypoglycemia or other safety aspects.

In addition to insulin treatment, one or a maximum of two OHAs were allowed (metformin, sulfonylurea, or dipetidyl peptidase (DPP)-IV inhibitors). The dosage of the OHAs remained stable during the study period. In case of postprandial blood glucose (PPG) values exceeding 11.1 mmol/L on two consecutive visits, treatment with prandial short-acting insulin was allowed.

Adherence to the insulin titration algorithm was confirmed by self-report of the physician. Insulin treatment adherence and adherence to OHA treatment of patients were measured by self-report and by evaluating the amounts of prescribed insulins and OHAs, respectively.

### Efficacy and safety endpoints

The primary efficacy endpoint of this study was a comparison of insulin glargine and NPH insulin used in BOT in terms of a composite DRQoL score, which was assessed at the end of each of the two treatment periods. The DRQoL consisted of a standardized and unweighted ITEQ score (Cronbach’s α = 0.93) [18], a PAID questionnaire score (Cronbach’s α = 0.86) [19, 20], and the mental health score of the SF-12® Health Survey [21]. The ITEQ was used to assess a range of factors, including leisure activities, sleep, weight control, and diabetes control, as well as general treatment satisfaction. The PAID questionnaire was designed to evaluate diabetes-specific emotional stress. The mental health score of the SF-12® included questions to indicate overall mental health as perceived by the patient. After converting the three sub-scores from these questionnaires to values in a 0–100 range, the composite score was calculated using the following formula: DRQoL = 1/3 * (ITEQ + (100 − PAID) + SF-12®). The range of values for DRQoL was 0–100, with 100 being the optimal value.

In addition to the composite DRQoL score, the individual questionnaire scores, and those from the EuroQol (EQ-5D) questionnaire [22, 23], and the Diabetes Treatment Satisfaction Questionnaire status version (DTSQs), were assessed. Each of the questionnaires was completed at baseline, crossover visit, and end of study, except for ITEQ which was not completed at baseline because participants included had no prior insulin treatment. Further secondary efficacy variables assessed the level of glycemic control by evaluating HbA1c values, FBG, and 7-point blood glucose profiles (determined by self-measured blood glucose readings). Additional parameters assessed as secondary variables were body weight, waist circumference, blood pressure, and lipids. Further secondary objectives and assessments included hypoglycemic events (symptomatic and/or severe), total daily insulin doses, and the patients’ treatment preference for insulin glargine vs. NPH insulin reported at the end of the study.

Safety endpoints were total number of serious adverse events (SAE) and adverse events (AE), including all forms of hypoglycemia, in particular, severe hypoglycemia (secondary efficacy endpoint), and localized pain, redness, or inflammation at the injection site. A 7-day follow-up period was used to ensure that all events that may have been related to treatment were included.

### Statistical considerations and analysis

In a previous cross-sectional study [16], different effect sizes of insulin glargine compared to NPH insulin in terms of SF-12®, PAID, and ITEQ scores were observed (d = 0.10, 0.11, and 0.29, respectively). The average effect size of all three scales was d = 0.166. Since the present study had a crossover design, in which each participant served as his/her own control, an effect size on the primary endpoint DRQoL of d = 0.20 was expected. Such an effect can be detected with 90 % power using a paired *t*-test with a significance level of 5 % and with 265 patient pairs. Considering a non-evaluable rate of 20 %, a total of 332 patients were to be enrolled in order to have 265 patients (completing both treatments) evaluable for the efficacy analysis.

The primary efficacy endpoint was evaluated by analysis of covariance (ANCOVA). The model included fixed effects for treatment, sequence, and period (treatment by sequence interaction), as well as a random effect to account for subjects within sequence. The Shapiro-Wilk test was applied to test the model assumption of normality of residuals at a critical level of 0.1. Statistical tests were performed at a significance level of α = 0.05.

Wherever possible, secondary endpoints were evaluated by analyzing changes from the start of the respective treatment period to its end. If applicable, treatment comparisons for secondary efficacy variables were made by the variance analytical approach described for the primary efficacy endpoint. The number of patients with at least one hypoglycemic event and the number of hypoglycemic events per patient year of insulin treatment was analyzed. Treatment differences in hypoglycemia rates were analyzed using the McNemar test. The same test was used to analyze response rates. For the questionnaires, single domain scores were summarized descriptively by treatment period. The total scores were also analyzed by ANCOVA. For the primary and secondary efficacy variables, two subgroups (by treatment sequence) were analyzed (within patient comparison): A) starting with insulin glargine and then switching to NPH insulin, and B) starting with NPH insulin and then switching to insulin glargine. In addition, treatment comparisons were performed focusing on each period separately (between patient comparisons).

Data entry, verification, and validation were carried out using SAS version 9.2.

## Results

### Patient disposition, demographics, and disease characteristics

**Fig. 2:**
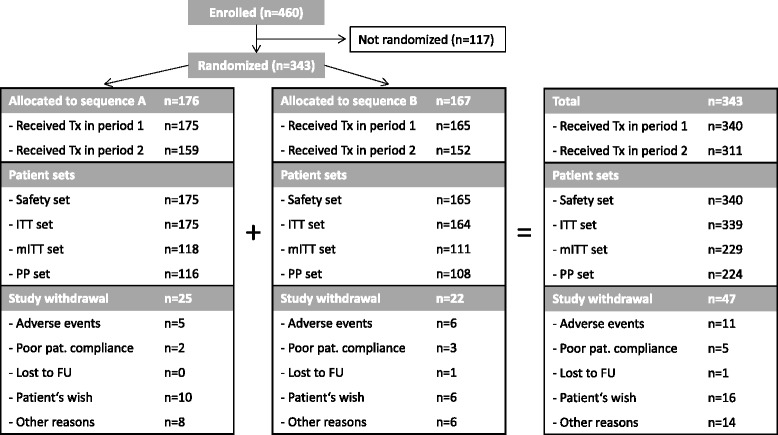
Patient disposition. No further data are available for patients not getting randomized

A total of 460 patients at 39 centers throughout Germany were screened for this study, of which 343 patients were randomized to either sequence A (n = 176) starting with insulin glargine or sequence B starting with NPH insulin (n = 167). A total of 151 (85.8 %) patients in sequence A and a total of 145 (86.8 %) patients in sequence B completed the study (Fig. 2). Of the 343 patients randomized, 340 received at least one dose of study drug (safety population), 339 had the required post-baseline efficacy data available (intention to treat; ITT), 229 had all values for the primary endpoint evaluation (modified intention to treat; mITT), and 224 had no major protocol violations (per-protocol; PP).

Patients comprising the ITT population had a mean age of 62.3 ± 9.0 years, 39.5 % were female, they had a high comorbidity burden, and the mean diabetes duration was 115.2 ± 71.0 months (equivalent to 9.6 ± 5.9 years), with no major difference between treatment sequences A and B (Table 1). Mean baseline HbA1c was 8.15 ± 0.72 %, and mean FBG level was 9.37 ± 2.19 mmol/L, with negligible differences between sequences.

**Table 1:** Patient and disease characteristics (ITT population)

	Sequence A (n = 175)	Sequence B (n = 164)	Total (n = 339)
Age in years, mean ± SD	61.9 ± 8.8	62.7 ± 9.2	62.3 ± 9.0
Female gender, %	38.3	40.9	39.5
Weight in kg, mean ± SD	90.1 ± 15.8	91.1 ± 15.1	90.5 ± 15.5
BMI in kg/m^2^, mean ± SD	30.9 ± 4.5	31.2 ± 4.7	31.0 ± 4.6
WC in cm, mean ± SD	106.4 ± 11.7	107.2 ± 11.4	106.8 ± 11.5
Diabetes duration in months, mean ± SD	115.1 ± 71.3	115.2 ± 71.0	115.2 ± 71.0
Diabetes duration in years, mean ± SD	9.6 ± 5.9	9.6 ± 5.9	9.6 ± 5.9
HbA1c in %, mean ± SD	8.17 ± 0.73	8.13 ± 0.72	8.15 ± 0.72
FBG in mmol/L, mean ± SD	9.24 ± 2.23	9.50 ± 2.16	9.37 ± 2.19
Time since first OHA treatment in months, mean ± SD	72.8 ± 62.2	67.3 ± 55.3	70.2 ± 58.9
Concomitant disease			
Cardiac disorders, %	28.0	23.8	26.0
Vascular disorders, %	86.3	88.4	87.3
Renal and urinary disorders, %	24.6	18.3	21.5
Concomitant oral medication			
Metformin, %	90.9	89.6	90.3
Sulfonylurea, %	57.7	54.9	56.3
DPP-IV inhibitors, %	22.3	26.2	24.2

Legend: Sequence A: starting with insulin glargine and then switching to NPH insulin; Sequence B: starting with NPH insulin and then switching to insulin glargine; SD, standard deviation; BMI, body mass index; WC, waist circumference; OHA, oral hypoglycemic agents; FBG, fasting blood glucose

### Primary endpoint (DRQoL scores)

On a 0–100 scale, the mean DRQoL scores in period 1 were 69.7 ± 8.45 with insulin glargine (sequence A) and 69.8 ± 9.81 with NPH insulin (sequence B). In treatment period 2, the mean scores were 69.4 ± 9.66 for insulin glargine (sequence B) and 70.1 ± 9.04 for NPH insulin (sequence A) (Table 2). When combining all data from both periods for each insulin treatment, mean DRQoL score was 69.6 ± 9.04 with insulin glargine and 70.0 ± 9.40 with NPH insulin. Neither an effect of treatment with insulin glargine vs NPH insulin (p = 0.31) nor a period effect (p = 0.96) nor a sequence effect (p = 0.76) was observed using an ANCOVA. However, it should be noted that model assumption of normally distributed residuals was not fulfilled. Within-patient comparisons did not show a treatment effect either for patients randomized to sequence A (p = 0.48) or for patients in sequence B (p = 0.46) (Table 2). Between-patient comparisons did not show a treatment effect either for the first treatment period (p = 0.97) or for the second treatment period (p = 0.55) (Table 2). Results were consistent in an analysis of the PP population as well as in a sensitivity analysis on the ITT population, where missing SF-12® mental health scores were replaced by the corresponding value reported at the baseline visit (data not shown).

**Table 2:** Summary of DRQoL scores [range 0–100] – between and within patient comparison of treatments (mITT population)

	End of period 1, mean ± SD	End of period 2, mean ± SD	*p*-value*
Sequence A	69.7 ± 8.45	70.1 ± 9.04	0.48
N = 118	(GLAR)	(NPH)	
Sequence B	69.8 ± 9.81	69.4 ± 9.66	0.46
N = 111	(NPH)	(GLAR)	
*p*-value **	0.97	0.55	

Legend: *Paired *t*-test was applied for comparison of treatments within each sequence (within patient comparison); **Unpaired *t*-test was applied for comparison of treatments in each period (between patient comparison); Sequence A: starting with insulin glargine (GLAR) and then switching to NPH insulin; Sequence B: starting with NPH insulin and then switching to insulin glargine

### Patient reported outcomes

For ITEQ, SF-12®, and EQ-5D, results were very similar between the two treatments and the two periods (Table 3). In line with the results of the primary endpoint, neither treatment effect, nor period effect, nor sequence effect was revealed by ANCOVA.

**Table 3:** Patient reported outcomes

	Baseline (Visit 2)	End of period 1	End of period 2	*p*-value*
ITEQ				
Sequence A	No insulin Tx	74.2 ± 11.8	73.3 ± 12.9	0.52
Sequence B	No insulin Tx	73.0 ± 13.7	73.2 ± 12.0	0.74
*p*-value**		0.42	0.97	
DTSQs Hyperglycemia				
Sequence A	4.2 ± 1.53	2.2 ± 1.57	2.3 ± 1.76	n.a.
Sequence B	4.2 ± 1.60	2.2 ± 1.73	2.1 ± 1.70	n.a.
*p*-value**		1.0	0.32	
DTSQs Hypoglycemia				
Sequence A	0.7 ± 1.32	1.2 ± 1.57	1.3 ± 1.68	n.a.
Sequence B	0.6 ± 1.15	1.0 ± 1.47	1.1 ± 1.52	n.a.
*p*-value**		0.25	0.29	
	Baseline (Visit 2)	∆ from baseline	∆ from end of period 1	*p*-value*
PAID				
Sequence A	21.3 ± 15.7	−5.1 ± 11.4	0.7 ± 10.4	<0.0001
Sequence B	23.3 ± 15.4	−4.4 ± 14.8	0.5 ± 11.7	0.0049
*p*-value**		0.61	0.89	
SF-12® mental health				
Sequence A	50.2 ± 9.34	0.5 ± 7.91	0.4 ± 7.56	0.68
Sequence B	49.8 ± 10.5	0.8 ± 9.21	−0.6 ± 8.59	0.24
*p*-value**		0.77	0.34	
SF-12® physical health				
Sequence A	50.5 ± 9.06	0.5 ± 7.94	−0.3 ± 8.04	n.a.
Sequence B	49.4 ± 9.20	0.9 ± 7.99	0.6 ± 7.29	n.a.
*p*-value**		0.70	0.36	
EQ-5D descriptive				
Sequence A	0.89 ± 0.16	−0.009 ± 0.1727	−0.005 ± 0.1787	0.91
Sequence B	0.900 ± 0.1682	0.001 ± 0.1606	−0.009 ± 0.1637	0.88
*p*-value**		0.62	0.85	
EQ-5D VAS				
Sequence A	0.867 ± 0.1730	−0.000 ± 0.1646	0.001 ± 0.1557	n.a.
Sequence B	0.862 ± 0.1819	0.009 ± 0.1655	−0.013 ± 0.1566	n.a.
*p*-value**		0.64	0.45	
DTSQs				
Sequence A	27.9 ± 7.72	3.2 ± 8.04	−0.8 ± 6.38	<0.0001
Sequence B	27.2 ± 7.37	2.1 ± 7.36	0.3 ± 6.92	0.0420
*p*-value**		0.22	0.16	

Legend: * Paired *t*-test was applied for comparison of treatments within sequence; ** Unpaired *t*-test was applied for comparison of treatments in periods; Sequence A: starting with insulin glargine (GLAR) and then switching to NPH insulin; Sequence B: starting with NPH insulin and then switching to insulin glargine (GLAR)

For the PAID questionnaire, no treatment effect (p = 0.71) and no sequence effect (p = 0.83), but a period effect (p < 0.0001) was revealed. Values decreased during period 1 for both insulin types, indicating reductions in diabetes-specific emotional distress (−5.1 ± 11.4 for sequence A with insulin glargine, −4.4 ± 14.8 for sequence B with NPH insulin. Only minor further changes occurred during period 2 for both treatments (Table 3).

In terms of treatment satisfaction (DTSQs), ANCOVA indicated a trend towards a treatment effect (p = 0.071), but no sequence effect (p = 0.89). A highly significant period effect (p < 0.0001) was also revealed. As shown in Table 3, scores increased by 3.2 ± 8.04 for sequence A with insulin glargine and 2.1 ± 7.36 for sequence B with NPH insulin in treatment period 1, showing only minor further changes during treatment period 2 (−0.8 ± 6.4 for sequence A with NPH insulin and +0.3 ± 6.9 for sequence B with insulin glargine). There were no differences between the treatments in DTSQs subscales focusing on hyper- or hypoglycemia (Table 3).

### Glucose metabolism and body weight

**Fig. 3:**
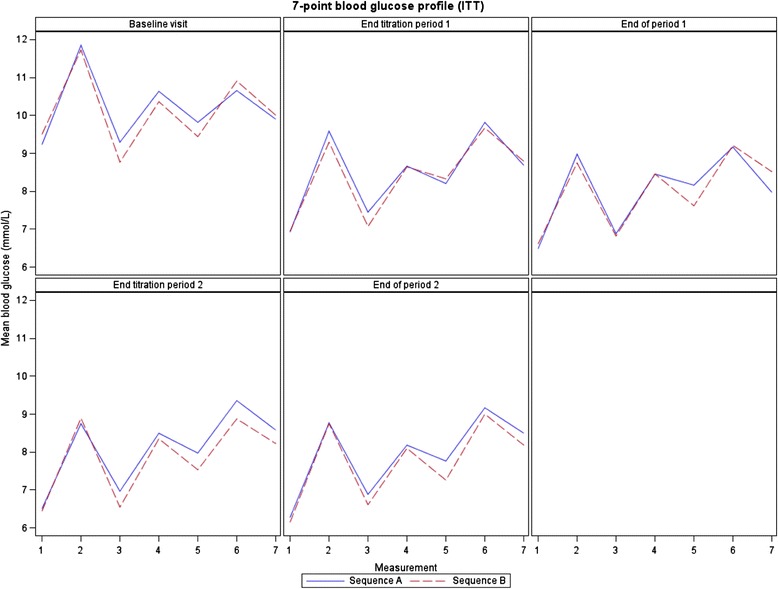
7-point blood glucose profile (ITT). Meaurements: 1-before breakfast, 2-2 h after breakfast, 3-before lunch, 4-2 h after lunch, 5-before dinner, 7-before sleeping. Source: bgprofcurve.sas[SVN:20879] Date Extract: 08FEB2013 Table Generation: 10JUNE2013 15:21

Regarding the effects on glucose metabolism, no differences between the two insulin types were observed (Tables 4, 5). Mean HbA1c values decreased by 1.17 % in the first treatment period for both insulin glargine and NPH insulin. In the second period, HbA1c values increased slightly compared to the crossover visit (0.21 % and 0.09 % in sequences A and B, respectively). No treatment effect (p = 0.65) or sequence effect (p = 0.17), but a period effect (p < 0.0001) on HbA1c levels was revealed by ANCOVA. Similarly, mean FBG was reduced in the first period compared to baseline, and then reduced a little further in the second period, with no significant differences between treatments. However, in terms of achieving the target of FBG ≤ 5.6 mmol/L, response rates were higher for patients receiving insulin glargine compared to NPH insulin (32.4 and 25.4 %, respectively; p = 0.02). For HbA1c levels, response rates were numerically although not significantly better for patients receiving insulin glargine in comparison to NHP insulin, with target HbA1c ≤ 7.0 % rates being 54.0 and 51.9 %, respectively (p = 0.64), and ≤ 6.5 % rates being 26.3 and 24.5 %, respectively (p = 0.69). Mean 7-point blood glucose profiles are displayed in Fig. 3. In terms of changes in body weight, neither a treatment effect (p = 0.97), nor a period effect (p = 0.57), nor a sequence effect (p = 0.84) was revealed by ANCOVA. Slight increases were observed during each period (insulin glargine: 0.40 kg in period 1 and 0.73 kg in period 2; NPH insulin: 0.51 kg in period 1 and 0.67 kg in period 2).

**Table 4:** Fasting blood glucose and insulin dose

	Baseline (Visit 2)	End of period 1	End of period 2
FBG (before breakfast) in mmol/L, mean ± SD			
Sequence A	9.24 ± 2.23	6.48 ± 1.66	6.27 ± 1.32
Sequence B	9.50 ± 2.16	6.60 ± 1.82	6.15 ± 1.42
*p*-value*		0.55	0.46
Daily insulin doses in U, mean ± SD			
Sequence A	0	24.9 ± 20.3	28.7 ± 20.1
Sequence B	0	24.8 ± 15.7	26.0 ± 17.0
*p*-value*		0.96	0.21
Insulin doses by bodyweight in U/kg, mean ± SD			
Sequence A	0	0.27 ± 0.20	0.31 ± 0.21
Sequence B	0	0.27 ± 0.15	0.28 ± 0.16
*p*-value*		0.88	0.13

Legend: * Unpaired *t*-test was applied for comparison of treatments in periods; Sequence A: starting with insulin glargine and then switching to NPH insulin; Sequence B: starting with NPH insulin and then switching to insulin glargine

**Table 5:** HbA1c and body weight

	Baseline (Visit 2)	∆ from baseline	∆ from end of period 1	*p*-value*
HbA_1c_ in %, mean ± SD				
Sequence A	8.17 ± 0.73	−1.17 ± 1.05	0.21 ± 0.58	<0.0001
Sequence B	8.13 ± 0.72	−1.17 ± 0.93	0.09 ± 0.63	<0.0001
*p*-value**		0.97	0.10	
Body weight in kg, mean ± SD				
Sequence A	90.1 ± 15.8	0.40 ± 3.31	0.67 ± 2.51	0.70
Sequence B	91.0 ± 15.13	0.51 ± 3.34	0.73 ± 3.10	0.68
*p*-value**		0.77	0.86	

Legend: * Paired *t*-test was applied for comparison of treatments within sequence; ** Unpaired *t*-test was applied for comparison of treatments in periods; Sequence A: starting with insulin glargine and then switching to NPH insulin; Sequence B: starting with NPH insulin and then switching to insulin glargine

### Insulin use

Mean daily insulin glargine dose was 17.9 ± 11.5 U at the end of the titration phase and was further increased by 7.3 ± 15.3 U during period 1. Similar values were obtained for NPH insulin (17.4 ± 10.7 and 7.6 ± 9.5 I.U., respectively). At the end of the titration phase of period 2, mean daily insulin glargine dose was 22.4 ± 14.6 and was further increased by 3.6 ± 6.6 U. Respective values obtained for NPH insulin were 23.3 ± 16.9 with an increase of 5.5 ± 8.4 I.U. ANCOVA results for change of insulin dose from end of titration to end of period revealed a small treatment effect (p = 0.041; increase in dose for NPH insulin was more pronounced than for insulin glargine) and a period effect (p < 0.0001; increase in dose was more pronounced in period 1 than in period 2 for both treatments), but no sequence effect (p = 0.56). At the end of period 1, mean insulin doses were 24.9 ± 20.3 U for insulin glargine and 24.8 ± 15.7 I.U. for NPH insulin, and at the end of period 2, the corresponding insulin doses were 26.0 ± 17.0 U and 28.7 ± 20.1 I.U. Respective values for insulin doses per kilogram bodyweight (kg BW) were 0.27 ± 0.20 U/kg BW at the end of period 1 and 0.28 ± 0.16 U/kg BW at end of period 2 for insulin glargine and 0.27 ± 0.15 I.U./kg BW and 0.31 ± 0.21 I.U./kg BW, respectively, for NPH insulin, showing no statistically significant differences (Table 4).

With regard to treatment preference, more patients continued the insulin treatment they received at the end of the study than wished to change (at the end of period 2). However, more patients changed from NPH insulin back to insulin glargine at the end of the study than vice versa. Thus, in sequence A, 47.4 % of patients continued on NPH insulin after the study, while 39.4 % switched back to insulin glargine. In sequence B, 67.7 % continued on insulin glargine and only 17.7 % switched back to NPH insulin. The odds ratio of switching back is 3.03; 95 % CI, 1.79 - 5.20; p < 0.001 in favor of insulin glargine.

### Hypoglycemia outcomes

The proportions of patients with hypoglycemia (defined as events in conjunction with a blood glucose measurement of <3.1 mmol/L) were 17.7 % during treatment with insulin glargine and 15.8 % during treatment with NPH insulin (Table 6). A total of 21 patients (6.4 %) experienced nocturnal hypoglycemia events during insulin glargine treatment and 24 patients (7.4 %) during NPH insulin treatment. Using a threshold of <3.9 mmol/L, the rates of patients with overall hypoglycemia, daytime hypoglycemia, and nocturnal hypoglycemia were 25.1 %, 20.5 %, and 7.6 %, respectively, during treatment with insulin glargine; and 23.2 %, 18.3 %, and 10.8 %, respectively, during treatment with NPH insulin. Two patients, both in the NPH insulin group, reported events that fulfilled the criteria for severe hypoglycemia (0.6 % in the NPH insulin group). Numbers of hypoglycemic events per patient year of insulin treatment resulted in comparable rates of hypoglycemia in either group, confirmed by blood glucose thresholds of <3.1 and <3.9 mmol/L. The mean number of events per patient year was 0.87 (95 % CI, 0.73–1.04) for insulin glargine and 0.81 (95 % CI, 0.67–0.97) using a threshold of <3.1 mmol/L. The corresponding results using a threshold of <3.9 mmol/L were 1.43 (95 % CI, 1.24–1.64) for insulin glargine and 1.46 (95 % CI, 1.27–1.67) for NPH insulin (Table 6). The evaluation of hypoglycemic events using a threshold of < 3.1 mmol/L separated for each period (Table 7) resulted in the following: in **period 1** the proportion of patients with overall hypoglycemia was 18.3 % during treatment with insulin glargine and 15.2 % during treatment with NPH insulin (OR = 1.24; 95 % CI, 0.70 – 2.21). In **period 2** the proportion of patients with overall hypoglycemia was 17.1 % during treatment with insulin glargine and 16.4 % during treatment with NPH insulin (OR = 1.06; 95 % CI, 0.58 – 1.92). Similar results were obtained using a threshold < 3.9 mmol/L with a proportion of patients with hypoglycemia of 25.1 % vs. 22.6 % (OR 1.15; 95%CI 0.70-1.90) in period 1 during treatment with insulin glargine vs. NPH insulin and 25.0 % vs. 23.9 % (OR 1.06; 95%CI 0.63-1.78) in period 2.

**Table 6:** Hypoglycemia Outcomes (ITT population)

	Episodes per patient year		Hypoglycemia rates		
Hypoglycemia	Insulin Glargine mean (95 %CI)	NPH insulin mean (95 %CI)	Insulin Glargine % (95 %CI)	NPH insulin % (95 %CI)	*p*-value*
	(N = 327)	(N = 323)	(N = 327)	(N = 323)	
Total exposure time in years^a^	144.8	144.5			
Threshold <3.1 mmol/L					
Overall hypoglycemia	0.87 (0.73-1.04)	0.81 (0.67-0.97)	17.7 (13.8-22.3)	15.8 (12.0-20.2)	0.2492
Daytime hypoglycemia	0.59 (0.48-0.73)	0.49 (0.38-0.62)	12.8 (9.4-17.0)	12.1 (8.7-16.1)	0.4751
Nocturnal hypoglycemia	0.28 (0.20-0.38)	0.32 (0.23-0.42)	6.4 (4.0-9.6)	7.4 (4.8-10.9)	0.7150
Threshold <3.9 mmol/L					
Overall hypoglycemia	1.43 (1.24-1.64)	1.46 (1.27-1.67)	25.1 (20.5-30.1)	23.2 (18.7-28.2)	0.2922
Daytime hypoglycemia	1.02 (0.86-1.20)	0.91 (0.76-1.08)	20.5 (16.2-25.3)	18.3 (14.2-22.9)	0.2253
Nocturnal hypoglycemia	0.41 (0.31-0.53)	0.55 (0.44-0.69)	7.6 (5.0-11-1)	10.8 (7.7-14.7)	0.1699
Severe hypoglycemia^b^	0.00 (NE-0.03)	0.01 (0.00-0.05)	0.0 (0.0-1.1)	0.6 (0.1-2.2)	NA

Legend: *McNemar test applied; ^b^one daytime event for each type of insulin; ^a^calculated by adding the exposure time of each patient to each type of insulin

**Table 7:** Hypoglycemia Outcomes by treatment phase (ITT population)

	Period 1			Period 2		
Hypoglycemia	Glar (N = 175) %	NPH (N = 164) %	OR (95%CI)	Glar (N = 152) %	NPH (N = 159) %	OR (95%CI)
Threshold <3.1 mmol/L						
Overall hypoglycemia	18.3	15.2	1.24 (0.70-2.21)	17.1	16.4	1.06 (0.58-1.92)
Daytime hypoglycemia	12.6	9.8	1.33 (0.67-2.63)	13.2	14.5	0.90 (0.47-1.71)
Nocturnal hypoglycemia	8.0	8.5	0.93 (0.43-2.02)	4.6	6.3	0.72 (0.27-1.94)
Threshold <3.9 mmol/L						
Overall hypoglycemia	25.1	22.6	1.15 (0.70-1.90)	25.0	23.9	1.06 (0.63-1.78)
Daytime hypoglycemia	20.0	15.9	1.33 (0.76-2.32)	21.1	20.8	1.02 (0.59-1.76)
Nocturnal hypoglycemia	9.1	12.2	0.72 (0.36-1.45)	5.9	9.4	0.60 (0.26-1.43)

### Safety

The overall profile of adverse events (AEs) was similar for the two insulin treatments. Overall, 46.2 % and 43.2 % of the patients experienced at least one AE during treatment with insulin glargine and NPH insulin, respectively. Serious AEs were reported for 7.3 % and 5.2 % of the patients, respectively.

Four patients died during the study: three patients during treatment with insulin glargine and one patient during treatment with NPH insulin. None of the AEs leading to death were considered as possibly related to the investigational drug by the investigator. The reported reasons for death were multiple organ failure, pancreatic cancer, cardiovascular failure, and ‘natural’ death.

A total of 12 AEs leading to discontinuation were reported for 6 patients (1.8 %) during treatment with insulin glargine. During NPH insulin treatment a total of 6 AEs leading to discontinuation were reported for 5 patients (1.5 %).

## Discussion

The principal aim of this study was to perform an intra-individual comparison of insulin glargine and NPH insulin with regard to patient reported outcomes such as treatment satisfaction, quality of life, and diabetes-specific emotional distress.

This randomized, controlled study was initiated owing to the scarcity of data regarding PROs after BOT initiation with insulin glargine or NPH insulin in type 2 diabetes patients. By using this crossover design, each patient could serve as his/her own control, allowing for direct comparison between the effects of the two drugs on quality of life. The DRQoL composite score used was designed to allow for a wide variety of PROs to be evaluated simultaneously. The ITEQ includes questions regarding lifestyle and general treatment satisfaction, the PAID questionnaire assesses diabetes-specific emotional distress, and the SF-12® provides information regarding the overall mental health of the patient. It was found that there were no statistically significant differences in DRQol score when making within-patient or between-patient comparisons. Furthermore, negligible differences were identified between the two treatment periods or between sequence A and sequence B. Of the previous evaluations of the effect of insulin glargine and NPH insulin on quality of life, none have reported highly significant differences between the two drugs. Hauner *et al.* described a retrospective, non-interventional, cross-sectional study (LIVE-DE) [16], where 1,602 patients (982 on insulin glargine, 620 on NPH insulin) were assessed in terms of quality of life using the ITEQ, PAID, SF-12®, and DTSQs. Patients treated with insulin glargine achieved statistically significant higher scores on the ITEQ, and physical subscale of the SF-12® questionnaire, with a trend towards superior scores found for the DTSQs, PAID, and mental health subscale of the SF-12®. However, retrospective, observational data such as those of the LIVE-DE study are prone to bias because of no random treatment allocation, and selection bias introduced by only being able to document those with a complete follow-up. Furthermore, BOT was only used in 43.0 % of insulin glargine and 16.3 % of NPH insulin patients, with a basal-bolus strategy being used in the majority of the remaining cases. In the present study, no differences were found in ITEQ, SF-12®, or EQ-5D scores when comparing treatments, periods, or sequences. In terms of the DTSQs score, a trend was apparent towards a higher score for patients receiving insulin glargine; however, the only statistically significant difference identified was when comparing the two treatment periods. Improved treatment satisfaction was evident at the end of period 1, but only slight further changes were found after switching to the other treatment.

In an open-label, 24-week, randomized controlled trial, Eliaschewitz *et al.* investigated differences in treatment satisfaction between patients randomized to treatment with glimepiride in combination with either insulin glargine or NPH insulin [8]. They observed better treatment satisfaction with insulin glargine (p < 0.02), as assessed using the DTSQ change version (DTSQc). Witthaus *et al.* reported on a 1-year, multicenter, open-label clinical study in 570 patients with type 2 diabetes that were randomized to receive either insulin glargine or NPH insulin, both in combination with oral agents [15, 24]. Patients completed the DTSQc and psychological Well Being Questionnaire (W-BQ) at baseline and at regular periods up to a year. Treatment satisfaction improved significantly (p < 0.01) in both groups, showing a non-significant tendency to be greater with insulin glargine vs. NPH insulin (p = 0.0634).

Bradley *et al.* [25] investigated the responsiveness of the DTSQc used in the trials by Eliaschewitz *et al.* and by Witthaus *et al.* [8, 15, 24], and compared it to the original DTSQs which we used in our trial (as a secondary endpoint). It was shown that, unlike in type 1 diabetes patients, no major effect of treatment was seen for patients with type 2 diabetes. Based on their results they concluded that benefits attributable to glargine, which would not be revealed by the DTSQs alone, became apparent on use of the DTSQc when used with people scoring at or near floor at baseline. This finding may explain why greater differences in treatment satisfaction were reported in the other trials than in the one presented here. Another point of note is that glucose metabolism at baseline was much poorer in the Eliaschewitz study in comparison to our own. This detail is of relevance because patients scored quite highly on the SF-12® and the PAID questionnaires at baseline in the present study, potentially making improvements harder to detect.

Based on the treatment algorithm with dedicated titration and concomitant pharmacotherapy, metabolic control was almost identical between treatment groups in our study. HbA1c levels strongly decreased during period 1, but slightly increased during period 2 for both groups. This increase may be due to poorer treatment adherence in the second period, or it could indicate that titration algorithms were followed less rigorously. FBG levels significantly improved in both groups from baseline to the end of period 1, and then further decreased in period 2. No statistically significant differences in rates of hypoglycemia were evident. This finding is consistent with the results obtained by Witthaus *et al.* [15, 24], where the decrease in HbA1c and the proportion of patients experiencing hypoglycemia were similar in both groups, and where trends for improved treatment satisfaction with insulin glargine were non-significant. In the study published by Eliaschewitz *et al.* [8], while equivalence was found for the two treatment groups in terms of changes in HbA1c levels from baseline (p = 0.795), a 27 % lower relative risk (RR) of hypoglycemia was demonstrated for insulin glargine in comparison to NPH insulin (RR, 1.27; 95 % CI, 1.03–1.57). Furthermore, the proportion of patients that experienced nocturnal hypoglycemia was much higher in the NPH insulin group (RR, 1.22; 95 % CI, 1.09–1.37; p < 0.001). Greater rates of hypoglycemia in patients receiving NPH insulin in comparison to insulin glargine have been reported in a similar magnitude in other trials [9, 10, 26]. The observation that patient groups who showed differences in hypoglycemia also displayed differences in treatment satisfaction, and vice versa those not showing differences in hypoglycemia displayed similar levels, demonstrates the significant influence of hypoglycemic events on the quality of life of type 2 diabetes patients receiving BOT. This conclusion is supported by previous studies that have shown strong associations between rates of hypoglycemia and quality of life in patients with type 2 diabetes [27, 28].

A notable finding of our investigation is that at the end of the 48 weeks of treatment, 39.4 % of patients receiving NPH insulin during period 2 (sequence A) switched back to insulin glargine. In contrast, only 17.7 % of patients receiving insulin glargine during period 2 (sequence B) switched back to NPH insulin. This outcome may indicate a limitation of the three questionnaires used to determine the impact of insulin glargine on treatment choices and patient reported outcomes.

The crossover design of this study enabled us to perform intra-individual comparisons of endpoint variables, abolishing differences in patient groups such as those seen in LIVE-DE and the Eliaschewitz study [8, 16]. Within this context, we opted to not perform a washout phase, contrary to other studies [29, 30], because of constraints emerging from discontinuing insulin treatment for a period of two weeks and restarting it thereafter. For safety reasons we recommended patients in period 2 to receive approximately 80 % of the daily dose of the insulin given before the crossover. The lack of washout phase, on the other hand, bears the potential for a substantial carry-over effect from one treatment to the other, with a potential reduction in differences in target achievement and treatment satisfaction. Patients at baseline were insulin naïve, while at the start of period 2, they had received 24 weeks of insulin treatment. This aspect of the study design is likely to provide further confounding effects when comparing the questionnaire scores at the end of the two treatment periods.

Another limitation of the study is that the treatment target of FBG ≤ 5.6 mmol/L was only achieved in 32.4 % of patients receiving insulin glargine and in 25.4 % of patients receiving NPH insulin. This result is in contrast to the 74.6 % and 70.7 % of insulin glargine and NPH insulin treated patients, respectively, reaching the FBG ≤ 5.5 mmol/L target in the Eliaschewitz study [8]. There was also a difference in mean insulin dose at the end of these two studies, with lower doses being administered in the present investigation. The failure to reach titration goals in the majority of both treatment groups, along with the lower doses of insulin, has the potential to mask any advantages of glargine treatment with regard to the avoidance of hypoglycemia and perceived benefits from a patient perspective.

Concomitant OHA therapy was only weakly defined in our study protocol. Only patients receiving one or a maximum of two oral hypoglycemic drugs (metformin, sulfonylurea, or DPP-IV inhibitors) were allowed. Doses of these were kept stable (±20 %) throughout the study, except for cases of hypoglycemia, when they could be reduced. Moreover, additional prandial short-acting insulin was allowed in cases of PPG values exceeding 11.1 mmol/L on two consecutive visits. This latter aspect of the study design may have led patients with difficulties in maintaining their blood glucose levels to appear as being better controlled than in fact they were, reducing the differences observed between treatment strategies.

### Clinical implications

The results of the present investigation indicate that when leading to insufficient but equivalent glycemic control and only small differences in hypoglycemia rates, insulin glargine and NPH insulin BOT provide similar quality of life and treatment satisfaction with the instruments used in this study. Deliberate omission of insulin injections has been reported by substantial numbers of patients, naming interference in lifestyle and problems with injections to be factors associated with this [31]. Therefore, achieving optimal quality of life for patients who require insulin therapy is of great importance. Initiation of insulin treatment often distresses patients, many of them perceiving it to be a result of personal failure in terms of achieving glycemic control [12, 13, 32]. In addition, it is in fact a sign of disease progression, which could be quite depressing for many individuals. The results from our investigation suggest that neither of the two types of insulin investigated provided significantly greater reduction in diabetes-related emotional distress when starting insulin therapy.

Due to the higher number of patients not reaching target glycemic control, previously reported differences in incidence of hypoglycemia when comparing insulin glargine and NPH insulin were not shown in the present study. As this factor has been associated with a lower quality of life, it appears that controlling hypoglycemia may be the most important consideration when the physician is selecting the type of insulin to prescribe.

## Conclusions

This phase IV, crossover study was focused on the impact of treatment with insulin glargine versus NPH insulin on patients’ judgment in terms of treatment satisfaction and quality of life using PROs. After treatment of the patients with both insulin types for 24 weeks each, the study demonstrated that insulin glargine and NPH insulin yielded similar results on the primary evaluation criterion, DRQoL. The results illustrate that, in a patient population with suboptimal HbA1c and FBG levels at baseline, with equivalent glycemic control, differences in the PRO questionnaires tested are negligible when comparing insulin glargine and NPH insulin.
